# Clinical, Anatomical, and Pathological Features in the Three Variants of Primary Progressive Aphasia: A Review

**DOI:** 10.3389/fneur.2018.00692

**Published:** 2018-08-21

**Authors:** Maxime Montembeault, Simona M. Brambati, Maria Luisa Gorno-Tempini, Raffaella Migliaccio

**Affiliations:** ^1^INSERM U 1127, CNRS UMR 7225, Sorbonne Universités, and Université Pierre et Marie Curie-Paris 6, UMR S 1127, Institut du Cerveau et de la Moelle Épinière (ICM), FrontLab, Paris, France; ^2^Centre de Recherche de l'Institut Universitaire de Gériatrie de Montréal, Montréal, QC, Canada; ^3^Département de Psychologie, Université de Montréal, Montréal, QC, Canada; ^4^Memory and Aging Center, University of California at San Francisco, San Francisco, CA, United States; ^5^Department of Neurology, Institut de la Mémoire et de la Maladie d'Alzheimer (IM2A), Paris, France

**Keywords:** primary progressive aphasia, nonfluent/agrammatic variant, semantic variant, logopenic variant, language, brain connectivity

## Abstract

Primary progressive aphasias (PPA) are neurodegenerative diseases clinically characterized by an early and relatively isolated language impairment. Three main clinical variants, namely the nonfluent/agrammatic variant (nfvPPA), the semantic variant (svPPA), and the logopenic variant (lvPPA) have been described, each with specific linguistic/cognitive deficits, corresponding anatomical and most probable pathological features. Since the discovery and the development of diagnostic criteria for the PPA variants by the experts in the field, significant progress has been made in the understanding of these diseases. This review aims to provide an overview of the literature on each of the PPA variant in terms of their clinical, anatomical and pathological features, with a specific focus on recent findings. In terms of clinical advancements, recent studies have allowed a better characterization and differentiation of PPA patients based on both their linguistic and non-linguistic profiles. In terms of neuroimaging, techniques such as diffusion imaging and resting-state fMRI have allowed a deeper understanding of the impact of PPA on structural and functional connectivity alterations beyond the well-defined pattern of regional gray matter atrophy. Finally, in terms of pathology, despite significant advances, clinico-pathological correspondence in PPA remains far from absolute. Nonetheless, the improved characterization of PPA has the potential to have a positive impact on the management of patients. Improved reliability of diagnoses and the development of reliable *in vivo* biomarkers for underlying neuropathology will also be increasingly important in the future as trials for etiology-specific treatments become available.

## Introduction

In 1892, Arnold Pick (1) first described a patient with a clinical history of progressive and isolated language deficits, along with mild memory impairment and progressive social dysfunction. Around the same time, Paul Sérieux (2) described a woman who presented with a progressive loss of word comprehension and in whom, contrary to Pick's patient, memory and intelligence were initially preserved. When this patient died in 1897, Jules Déjerine examined his brain, discovering neuronal loss and cortical atrophy in bilateral temporal regions (2). More recently, Marsel Mesulam had the opportunity to examine the cell and myelin preparations of Sérieux's patient, finding no evidence of either senile plaques or neurofibrillary tangles (3). For this reason, he considered this patient the closest prototypical example of the syndrome now known as primary progressive aphasia (PPA).

In the modern literature, the first systematic description of a series of PPA cases was published in 1982 by Marsel Mesulam (4). The disorder was characterized as a “slowly progressive aphasia without generalized dementia.” The six reported patients had very heterogeneous linguistic profiles, which did not completely fit the classic vascular aphasia models of Broca and Wernicke, thus suggesting the existence of several variants of PPA.

In the mid-1970s, Warrington (5), followed by Snowden (6), Hodges and their collaborators (7) described a progressive disorder of semantic memory that they termed “semantic dementia.” In 1996, Grossman (8) described a different form of progressive language disorder, termed “progressive nonfluent aphasia.” At the end of the 1990s, Neary and collaborators (9) proposed a classification for frontotemporal dementia (FTD, then used as a clinical term) that included semantic dementia and progressive nonfluent aphasia. However, the definition of progressive nonfluent aphasia was very broad and likely included a variety of clinical syndromes. In 2004, a third subtype of PPA was described by Gorno-Tempini and colleagues (10), the “logopenic” variant of PPA (lvPPA). In 2011, an international group of PPA investigators (11) put forth new criteria that included these three main variants. These criteria are based on clinical features, along with neuroimaging, neuropathological, and genetic data, to allow homogeneous patient classification for research purposes. In this framework, patients must first meet the general PPA criteria proposed by Mesulam, in which a difficulty with language must be (1) the most prominent clinical feature, both at symptom onset and for the initial phases of the diseases, and (2) must be the principal cause of impaired daily living activities (12). The general clinical evaluation of PPA patients aims to identify the speech-language profile, showing impaired vs. spared speech-language skills, in order to identify the variant. In addition to the clinical features, the diagnosis can be further supported by neuroimaging (atrophy, hypometabolism, hypoperfusion), and pathology. Table 1 summarizes the clinical, anatomical, and biological features of the three main variants of PPA.

**Table 1:** Clinical, anatomical, and biological features of the three variants of PPA.

**Primary progressive aphasia variant**	**Clinical**	**Anatomical**	**Most common pathology**
Non fluent/agrammatic	Effortful speech, AOS, dysarthria, agrammatism	Left inferior frontal gyrus and insula	FTD-4R tau
Semantic	Impaired retrieval and comprehension of low frequency single words, semantic deficits for objects and people, surface dyslexia/dysgraphia	Bilateral anterior temporal lobe, usually left>right	TDP-43-C
Logopenic	Word-finding difficulty, sentence repetition/comprehension deficits and phonological dyslexia/dysgraphia	Left inferior parietal lobule and posterior superior temporal gyrus	AD

*AOS, Apraxia of speech; FTD, frontotemporal dementia; TDP-43, transactive response DNA-binding 43; AD, Alzheimer's disease*.

Although these three main variants do not account for all possible presentations of PPA, this classification is thought to capture most patients who do not a have a genetic form of the disease. It should be noted that, while correct clinical characterization allows accurate prediction of anatomical involvement, the correspondence between clinico-anatomical and molecular findings at pathology is only probabilistic. It reflects partially selective vulnerability of certain networks to certain neurodegenerative diseases (13).

Since the discovery and the development of diagnosis criteria for the PPA variants by the experts in the field in 2011, significant progress has been made in the understanding of PPA. While most neuroimaging studies on PPA had focused on regional atrophy, recent neuroimaging studies have focused on the impact of PPA on selective brain networks sustaining different language functions. Furthermore, recent longitudinal studies have allowed a better delineation of clinical and cerebral changes associated with the progression of the disease. This review aims to provide an overview and an update of the literature on each of the PPA variant in terms of their clinical, anatomical and pathological features. More specifically, in addition to reviewing the 2011 criteria, this review will provide an update on recent findings on the linguistic/cognitive manifestations of the disease and on their assessment. The latest neuroimaging studies will be reviewed with a specific focus on disconnection aspects of the disease. Issues and controversies associated with the diagnosis of PPA will be discussed, and possible avenues will be examined in the light of the most recent research.

## General background on pathology in PPA

From a pathological point of view, each PPA variant seems to correspond to a specific tissue pathology, and in some cases to a gene mutation. However, the clinico-pathological correspondence in PPA remains far from absolute, as will be discussed more specifically for each PPA variant in the following sections (Table 1).

One of the possible causes is the accumulation of pathological aggregates of tau protein. Tau protein is a highly soluble microtubule-associated protein (MAPT) and promotes microtubule polymerization and stabilization. Disorders in which tau pathology is considered the major contributing factor to neurodegeneration are referred to as “primary tauopathies” (e.g., fronto-temporal dementia tau related, FTD tau). Tau protein in the brain is heterogeneous due to alternative splice forms, as well as post-translational modifications, including phosphorylation (14). The terms “3R” and “4R” tau refers to the products of the alternative splicing of the MAPT gene, generating tau species with either three or four conserved ~32 amino acid repeats in the microtubule binding domain of tau protein (15). There is preferential accumulation of 3R or 4R tau in various tauopathies, providing a biochemical subclassification of the tauopathies. The MAPT gene is located on chromosome 17. Mutations in the MAPT gene induce the formation of abnormal normal tau protein inclusions, leading to abnormal functioning of these cells. Changes in tau protein induced by mutations can also decrease its effectiveness or increase its amount, which can lead to disease.

Another frequent pathological substrate is the deposits of transactive response DNA-binding protein 43 (TDP-43) which is a cellular protein encoded by the TARDBP gene. Four subtypes have been described (A, B, C, D), in some cases associated with genes mutations. For instance, type A (TDP-43-A) is associated in a proportion of cases with mutations in the progranulin (PGRN or GRN) gene (16). The PGRN gene is located on chromosome 17 and induces the production of the progranulin protein. Mutations in the PGRN gene reduce the production of progranulin and increase the neural aggregates of TDP-43. The progranulin helps cell growth, and the protein TDP-43 regulates the process of making proteins from DNA (expression). The TARDBP gene on chromosome 1, encoding the TDP-43 protein, is very rarely involved.

Finally, some patients (especially those with lvPPA) have an underlying AD pathology. AD is characterized by intracellular tau-associated neurofibrillary tangles and extracellular amyloid-β (Aβ)–associated plaques in the brain.

## Variants of primary progressive aphasia (PPA)

### Non fluent/agrammatic variant (nfvPPA)

#### Clinical manifestations

NfvPPA is a rare, early-onset neurodegenerative syndrome with a mean age of onset of ~60 years (17). The duration of survival is quite variable, ranging from 2 years in cases associated with amyotrophic lateral sclerosis to about 12 years in cases not associated with any motor disorder (18, 19).

The hallmark clinical features of nfvPPA are effortful speech and agrammatism. Effortful speech is characterized by slow, labored speech production, mainly due to a speech motor planning deficit, i.e., apraxia of speech (AOS) (20). Speech sound errors, consisting of distortions, deletions, substitutions, insertions, or transpositions of speech sounds are present. Distortions are considered as phonetic errors and are caused by AOS, while deletions, substitutions, insertions and transpositions are phonemic errors and can be caused by a motor speech impairment or a phoneme selection deficit (21, 22). However, there are some significant challenges in differentiating these two types of errors clinically, and both studies showing higher rates of phonetic errors (23, 24) or higher rates of phonemic errors (21, 22) in nfvPPA patients have been reported. Prosody is also typically affected in nfvPPA. Dysarthric features often co-occur with AOS, usually with mixed hypophonic and spastic features (20). Agrammatism is characterized by short, simple phrases, and omission of grammatical morphemes. Difficulties are present in language production (e.g., omission of articles, use of incorrect morphological endings), as well as in comprehension (e.g., difficulties in understanding complex syntactic structures, such as passives and relative clauses) (25). Patients with nfvPPA often use fewer verbs compared with healthy controls, in part because verbs play a critical part in syntactically structuring a sentence. They also have difficulty with verb naming and comprehension tasks (26).

With the progression of the disease, other cognitive deficits may emerge, including a decline in attentional resources and verbal working memory, as well as executive functions, episodic memory, praxis and behavioral symptoms (27, 28). General neurological examination is normal early in the disease course but extrapyramidal signs, and in many cases, a florid progressive supranuclear palsy syndrome (PSP-s) or corticobasal syndrome (CBS), can occur later in the disease course (24, 29–34) when the disease advances to SMA and subcortical regions, which might be due to the underlying FTD-4R pathology (see section Pathology). In cases in which language difficulties are very early accompanied by a clear extrapyramidal syndrome (for example, a generalized rigidity or tremor), the diagnosis of PPA is excluded. In these cases, even though the specific criteria for nfvPPA might be fulfilled, the general PPA criteria proposed by Mesulam are not fulfilled (12), given the predominance of the extrapyramidal syndrome and its impact on daily living activities. Therefore, clinically, these patients are diagnosed with PSP-s or CBS with speech/language features (35, 36). Conversely, mild deficits, such as mild limb apraxia or slowness in fine finger movements, do not exclude a diagnosis of PPA.

The current clinical criteria (11) for nfvPPA include at least one of the following core features: (1) Agrammatism in language production; (2) Effortful, halting speech with inconsistent speech sound errors and distortions (AOS); and at least 2 of 3 of the following other features: (1) Impaired comprehension of syntactically complex sentences; (2) Spared single-word comprehension; (3) Spared object knowledge.

#### Linguistic/cognitive assessment

Spontaneous speech in nfvPPA can be assessed using description of a picture, such as “The cookie theft” (37), “The picnic scene” from the Western Aphasia Battery (38), or a picture story such as “Frog, where are you?” (39). These connected speech samples may yield information about fluency, grammatical competence, and motor speech abilities, amongst others (23, 40). Previous studies have revealed that speech samples in nfvPPA are characterized speech sound errors, as well as slow rate, syntactic errors, and reduced complexity (21–23). Specific tests for the motor speech component, performed by an expert speech pathologist, are also highly recommended. These may include articulatory tasks of increasing difficulty, from simple phonation and production of single syllables (e.g., puh/puh/puh) to more complex diadochokinetic tasks (e.g., puh/tuh/kuh/), repetition of multisyllabic words (e.g., impossibility), and finally, sentence repetition [e.g., (41)].

Motor speech disorders may, in some cases, prevent the accurate assessment of agrammatism. One approach is to use sentence production tasks, such as the Northwestern Anagram Test, which requires to assemble individual word cards into a meaningful sentence (42). Another approach is to assess grammatical processing in comprehension, by asking patients to point to one of two pictures after hearing an auditorily presented sentence. In this task, accurate decoding of the grammatical structure of the sentence is required to select the correct picture. Conversely, in very early stages of disease, written language (such as a written description of a picture) can often reveal early, mild grammatical errors. Specific tests for word-comprehension and object knowledge, functions usually spared in the early phases, should also be systematically administrated.

#### Neuroimaging findings

**Figure 1:**
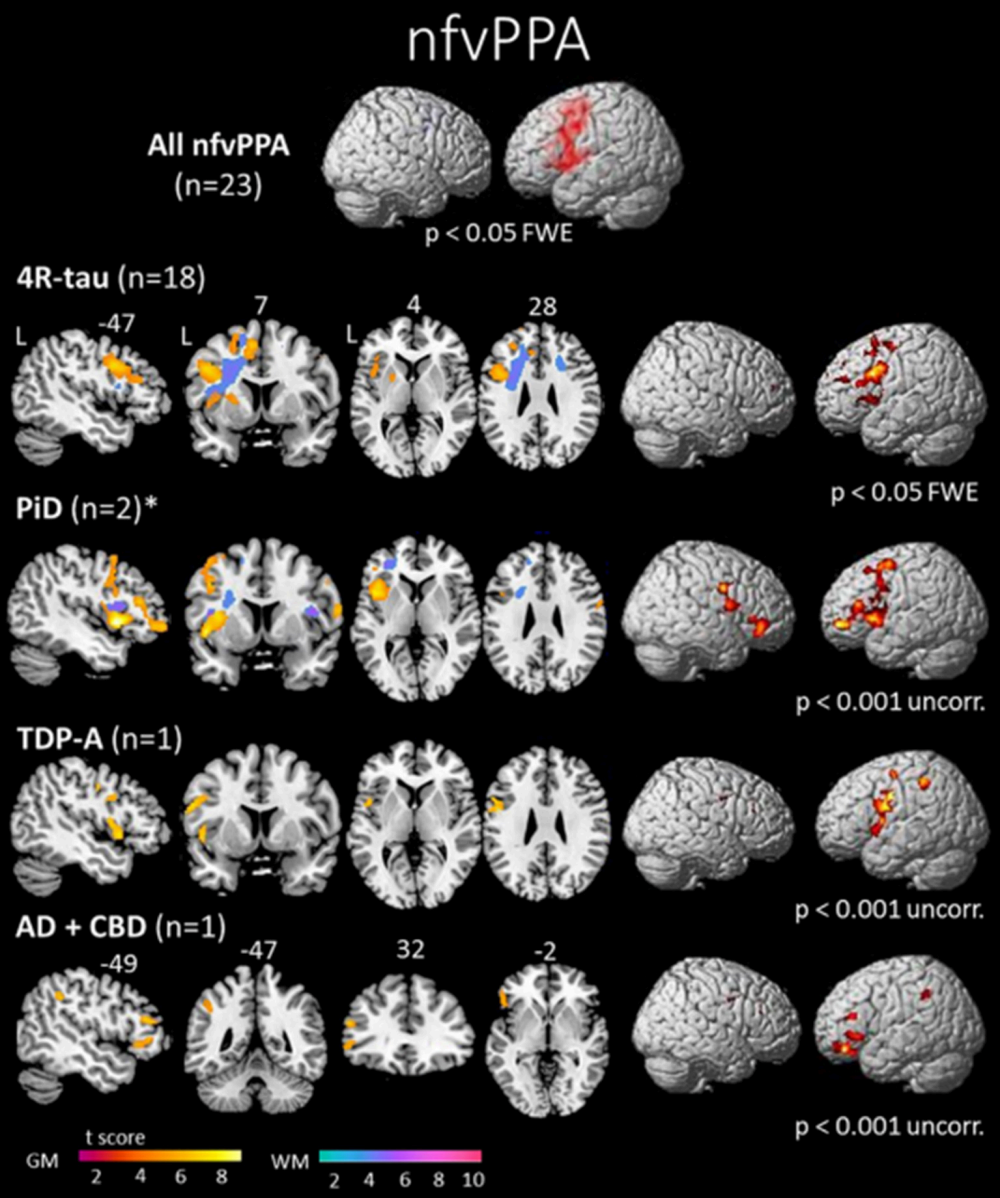
Areas of significant atrophy in a group of patients with nonfluent/agrammatic variant of primary progressive aphasia (nfvPPA) and then subdivided by pathological subgroups. The main areas of atrophy are mainly in the left inferior frontal gyrus, insula and premotor areas. No significant difference was observed in the comparison between nfvPPA-tau and nfvPPA-TDP-43 patients in this study. However, greater white matter damage has been reported in nfvPPA-tau cases (69). FWE, family wise error; 4R-tau, nfvPPA patients with FTD-tau with 4 repeats pathology; PiD, nfvPPA patients with Pick's disease pathology; TDP-A, nfvPPA patients with FTD-TDP-43 depositions type A pathology; AD + CBD, nfvPPA patients with Alzheimer's disease and corticobasal degeneration pathologies; GM, gray matter; WM, white matter; L, left [from (45), Permission to reproduce have been obtained from the copyright holders of this work].

The left inferior frontal gyrus (pars opercularis) is considered as the syndrome-specific epicenter in nfvPPA (43, 44). It is also associated with gray matter (GM) atrophy in the insula, premotor regions, SMA and striatum (Figure 1) (10, 45, 46). Syntactic processing deficits observed in these patients are associated with structural and functional abnormalities in the posterior part of inferior frontal gyrus (47). Some patients can become mute early in the course of the disease. This profile is associated with GM atrophy that is more prominent in the pars opercularis, extending into the left basal ganglia (48).

A few studies reported that AOS and agrammatism can occur separately in nfvPPA, affecting different subcomponents of the same brain network (49). In cases in which AOS presents as an isolated symptom, the term of “primary progressive apraxia of speech” has been applied (PPAOS). On one hand, patients with PPAOS, might show focal imaging abnormalities in the premotor cortex. On the other hand, patients with dominant agrammatic deficits show widespread involvement of premotor, prefrontal, temporal, and parietal lobes, as well as in the caudate nucleus and the insula (49). Nevertheless, in the most common presentation of nfvPPA, motor speech deficits are prevalent, but signs of agrammatism are also present. Atrophy in these cases includes premotor and posterior inferior frontal regions, progressing along the aslant tract to the supplementary motor area (SMA) complex and eventually to the basal ganglia and supramarginal gyrus (46). nfvPPA is therefore an example of a network disorder involving the circuit of regions and connections involved in speech production.

**Figure 2:**
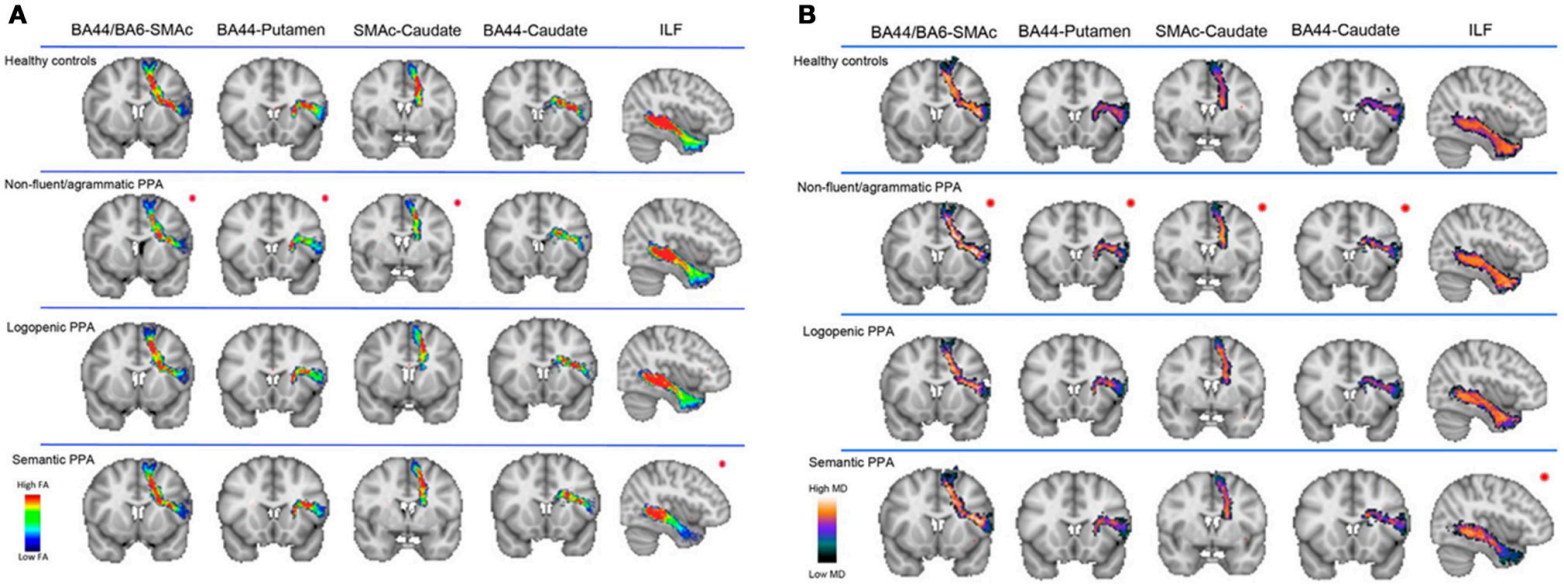
Speech production white matter tracts are shown in healthy controls, nfvPPA, lvPPA, and svPPA patients. Fractional anisotropy (FA) **(A)** and mean diffusivity (MD) **(B)** metrics were evaluated in four speech production tracts (BA44-SMAc, BA44-Putamen, SMAc-Caudate, and BA44-Caudate) and in the inferior longitudinal fasciculus (ILF). FA is a measure that quantifies the degree to which the diffusion of water molecules in WM fiber bundles is restricted to a specific direction (a higher FA value is associated with a better structural integrity). MD is a measure that quantifies the average degree of diffusion of water molecules in all directions (a lower MD value is associated with a better structural integrity). These results suggest that the white matter tracts connecting the speech production network are selectively damaged in nfvPPA. BA44, Broadmann Area 44; SMAc, Supplementary motor area complex; ILF, inferior longitudinal fasciculus. [from (46), Permission to reproduce have been obtained from the copyright holders of this work].

**Figure 3:**
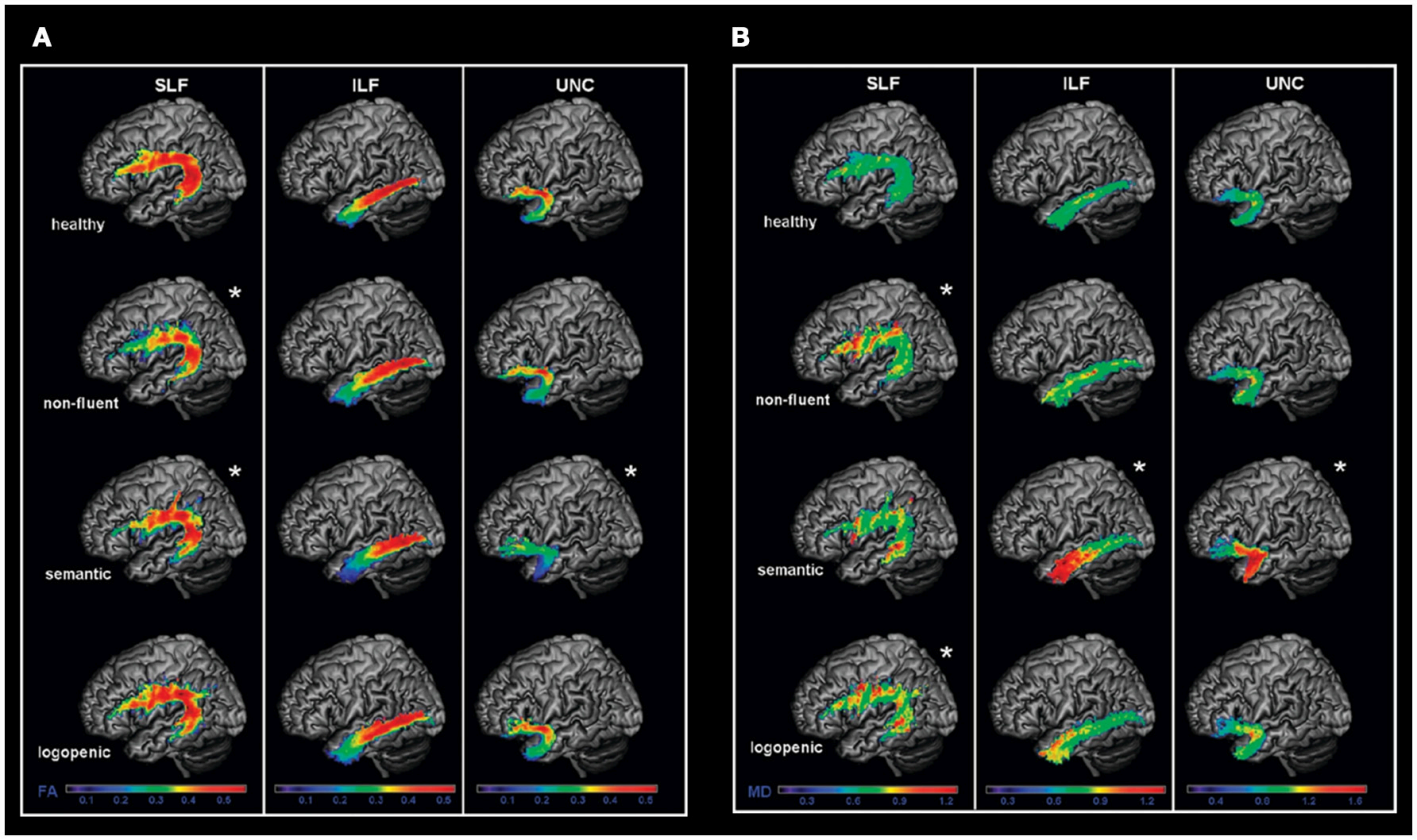
Left language-related white matter tracts are shown in healthy controls, nfvPPA, svPPA, and lvPPA patients. Fractional anisotropy (FA) **(A)** and mean diffusivity (MD) **(B)** metrics were evaluated in three tracts: the SLF (dorsal pathway), the ILF and UNC (ventral pathways). FA is a measure that quantifies the degree to which the diffusion of water molecules in WM fiber bundles is restricted to a specific direction (a higher FA value is associated with a better structural integrity). MD is a measure that quantifies the average degree of diffusion of water molecules in all directions (a lower MD value is associated with a better structural integrity). In nfvPPA, the main damage was observed in the dorsal pathway. In svPPA, the two ventral pathways as well as the temporal part of the dorsal pathway was damaged. In lvPPA, only the temporoparietal part of the dorsal pathway was damaged. SLF, Superior longitudinal fasciculus; ILF: inferior longitudinal fasciculus; UNC: uncinated fasciculus; FA, fractional anisotropy; MD, mean diffusivity [from (51), Permission to reproduce have been obtained from the copyright holders of this work].

Diffusion magnetic resonance imaging (MRI) techniques have shown that the dorsal language pathway of long-range white matter (WM) fibers connecting frontal, subcortical, and parietal areas are primarily involved in neurodegeneration in nfvPPA (46, 50) (Figure 2). This damage appears to be specific to this variant and is not observed in other PPA variants. WM damage in the dorsal pathway (superior longitudinal fasciculus) is also observed (Figure 3) (51–53). Consistently, a recent resting state fMRI study has demonstrated decreased functional connectivity between the left inferior frontal gyrus and the posterior middle temporal gyrus in nfvPPA, even in patients in which the atrophy is not severe (54).

Longitudinal GM atrophy changes in nfvPPA occur, 1 year after the first visit, in the left posterior frontal regions (often comprising inferior middle and superior gyri), supplementary motor area, insula, striatum, inferior parietal regions, and underlying WM (43, 55–57). Atrophy progresses to the supplementary motor complex region through the aslant tract. This tract is involved in the initiation and execution of movements, especially articulation. In nfvPPA, its integrity is associated with the number of distortion errors made by patients in spontaneous speech as well as with performance in a verbal fluency task (46, 58).

#### Pathology

NfvPPA is most commonly associated with a form of FTD-4R tau (45, 59–65). Other reports indicate TDP-43-A pathology in nfvPPA (45, 59, 66) and in some cases associated with progranulin (PGRN) or chromosome 9 open reading frame 72 (C9ORF72) gene mutations (67). Less frequently, AD pathology has also been reported in nfvPPA (45, 60, 61). However, a recent study investigating PPA patients with discordant amyloid status (i.e., nfvPPA with AD pathology) has suggested that most of these cases actually present mixed pathology (FTD tau pathology as primary pathologic diagnosis and AD pathology as contributing pathologic diagnosis) (68).

While progress has been made in understanding the underlying pathology in nfvPPA, some recent studies have also begun to characterize nfvPPA patients according to their underlying pathology. Clinically, it has been hypothesized that cases with predominant and isolated motor speech disorders would be associated with tau (24), while predominant agrammatism could predict TDP-43-A pathology. While similar GM damage have been observed in nfvPPA-tau and nfvPPA-TDP patients (Figure 1), greater WM damage has been observed in nfvPPA-tau cases (45, 69). Recently, further involvement of temporo-parietal regions beyond GM loss in the frontal lobe has been detected in a clinically heterogeneous group of TDP-43-A cases (64).

NfvPPA patients with underlying FTD-4R tau can also be further divided in nfv-PPA associated with progressive supranuclear palsy (PSP) and with corticobasal degeneration (CBD) anatomopathologies (56). At initial presentation, dysarthria and relatively selective WM atrophy appear typical of nfvPPA-PSP, while greater sentence comprehension deficits appear typical of nfvPPA-CBD. While these speech/language differences dissipate at a one-year follow-up, the progression of atrophy also allow to differentiate the two subgroups (56). In nfvPPA-PSP, atrophy spreads within the subcortical/brainstem motor system, which is consistent with greater oculomotors deficits and swallowing difficulty. In nfvPPA-CBD, atrophy progresses anteriorly in prefrontal regions, which is thought to generate greater working memory impairment and behavioral symptoms.

### Semantic variant (svPPA)

#### Clinical manifestations

SvPPA is characterized by a progressive and multimodal loss of semantic knowledge (70). Age at onset is variable, most often between 55 and 70 years (71). The duration of the disease is also variable and can range from 2 to 15 years, although patients typically survive 7–8 years after onset (72, 73). Studies of the prevalence and incidence of svPPA are relatively limited, but a recent epidemiology study estimated the prevalence of FTD at 10.8/100,000, with svPPA accounting for approximately one-third of these cases (74).

These patients progressively lose the meaning of words, and they usually present with severe, progressive anomia, and markedly impaired comprehension of single words (11, 30). In earlier stages, the loss of meaning of words, and subsequently, naming and single-word comprehension deficits, are more prominent for low frequency/familiarity items (e.g., “rhinoceros” vs. “dog”) (75). Often, the patients replace less frequent words with more familiar ones, typically using the superordinate category (e.g., “animal” for “cat”). Another salient aspect of the syndrome is the production of semantic paraphasias in naming (e.g., “brush” for “comb”). Anomia can also be observed in spontaneous speech that is often empty and not very informative (23, 76). In the early stages, inability to comprehend low-familiarity words can be the only symptom accompanying anomia, and patients frequently ask for the meaning of words. The progression of semantic deficits leads to impaired object recognition affecting all sensory modalities, including vision, touch, olfaction, and gustation (7, 70) [e.g., visual agnosia; (73)]. The ability to correctly identify objects is strongly influenced by familiarity with the object (e.g., “fork” is more familiar than “compass”) (77). Additionally, individuals with svPPA appear to have disproportionate difficulty understanding concrete concepts relative to abstract concepts (78–80). Rarely, cases have been described with greater, or even selective, deficits for people (81) and animals (82). Some patients demonstrate impairment in the recognition of faces, which stems from a loss of person knowledge (83) that is also familiarity-dependent (84).

In contrast, episodic memory is relatively preserved in svPPA, especially when tasks with minimal conceptual loading are used (85, 86). The intact performance on traditional non-conceptually loaded episodic memory tasks converges with the performance of svPPA patients on autobiographical memory tasks. Patients typically show relatively preserved recollection of recent autobiographical memory in the context of poorer remote autobiographical memory (known as the reverse temporal gradient or step-function), reflecting increased semanticisation of past events (87, 88). SvPPA patients have also difficulties in episodic future thinking (89, 90).

The loss of word meaning is also apparent in reading. Patients do not recognize words as whole entities, but rather adopt a phonological strategy, deriving pronunciations using letter-sound conversion. As a result, irregular words are pronounced as if they are regular (“yatsht” for “yacht”), a phenomenon called surface dyslexia (91, 92). A similar pattern of selective impairment for irregular words is observed in spelling (surface dysgraphia).

Behavioral abnormalities are typically present in mid-late phases, including disinhibition, irritability, and food taste changes (e.g., preference for sweet foods). Lack of empathy, mental inflexibility, and compulsions - including clockwatching and intense interest in jigsaws—are also frequently noted (93–95). Almost 50% of svPPA patients report experiencing somatic symptom disorder as misidentification and preoccupation with normal bodily sensations such as hunger, bladder filling, borborygmi, rhinorrhea, and reflux; excessive concern over the incompletely understood meaning or source of pain or other symptoms; and Cotard syndrome or the delusion that unidentified somatic symptoms signify death or deterioration (96). This inability to read and name somatic sensations, or “alexisomia,” results in disproportionate and persistent concern about somatic sensations with consequent significant disability (96).

It has been demonstrated that non-right-handedness is overrepresented in svPPA patients, at nearly twice the prevalence of the general population. Left-handedness has been described as a proxy for atypical brain hemispheric lateralization (97).

The current diagnostic guidelines (11) identify anomia and single-word comprehension deficits as core features, both essential for diagnosis. At least 3 of the following other diagnostic features must also be present: (1) impaired object knowledge, particularly for low- frequency or low-familiarity items; (2) surface dyslexia or dysgraphia; (3) spared repetition; and 4. spared speech production (grammar and motor speech).

#### Linguistic/cognitive assessment

Language assessment of svPPA includes tests of confrontation naming, in which the patient is asked to retrieve the word in response to a picture [e.g., the Boston Naming test (98)]. Object and person knowledge are also examined using tests of semantic associations, gesture-object matching, and sound-picture matching tasks. The popular Pyramids and Palm Trees Test (99) is a semantic association task that measures the capacity to access detailed semantic information about words and pictures necessary for the identification of the relationships that conceptually link two perceptually and functionally distinct entities. The loss of concepts can be also tested using other types of stimuli, including sounds, foods, and odors. Famous faces and buildings naming tasks, as well as semantic knowledge tasks are also very sensitive with svPPA patients (100).

Reading and spelling of regular and irregular words are also tested in order to identify surface dyslexia and dysgraphia. Spontaneous speech should also be assessed in svPPA. Differently from nfvPPA, the spontaneous speech should not present AOS and the syntactic structure should be preserved. On the other side, svPPA present increased use of highly familiar words, anomia characterized by long pauses and use of general words (such as “thing”) for identifying the items displayed on the image.

Episodic memory tests based on non-linguistic stimuli (such as Rey Complex Figure) should be administered to exclude the presence of major episodic memory deficits, especially at early stages of the disease. Repetition and syntactic comprehension tests should be evaluated as an exclusion criterion.

#### Neuroimaging findings

**Figure 4:**
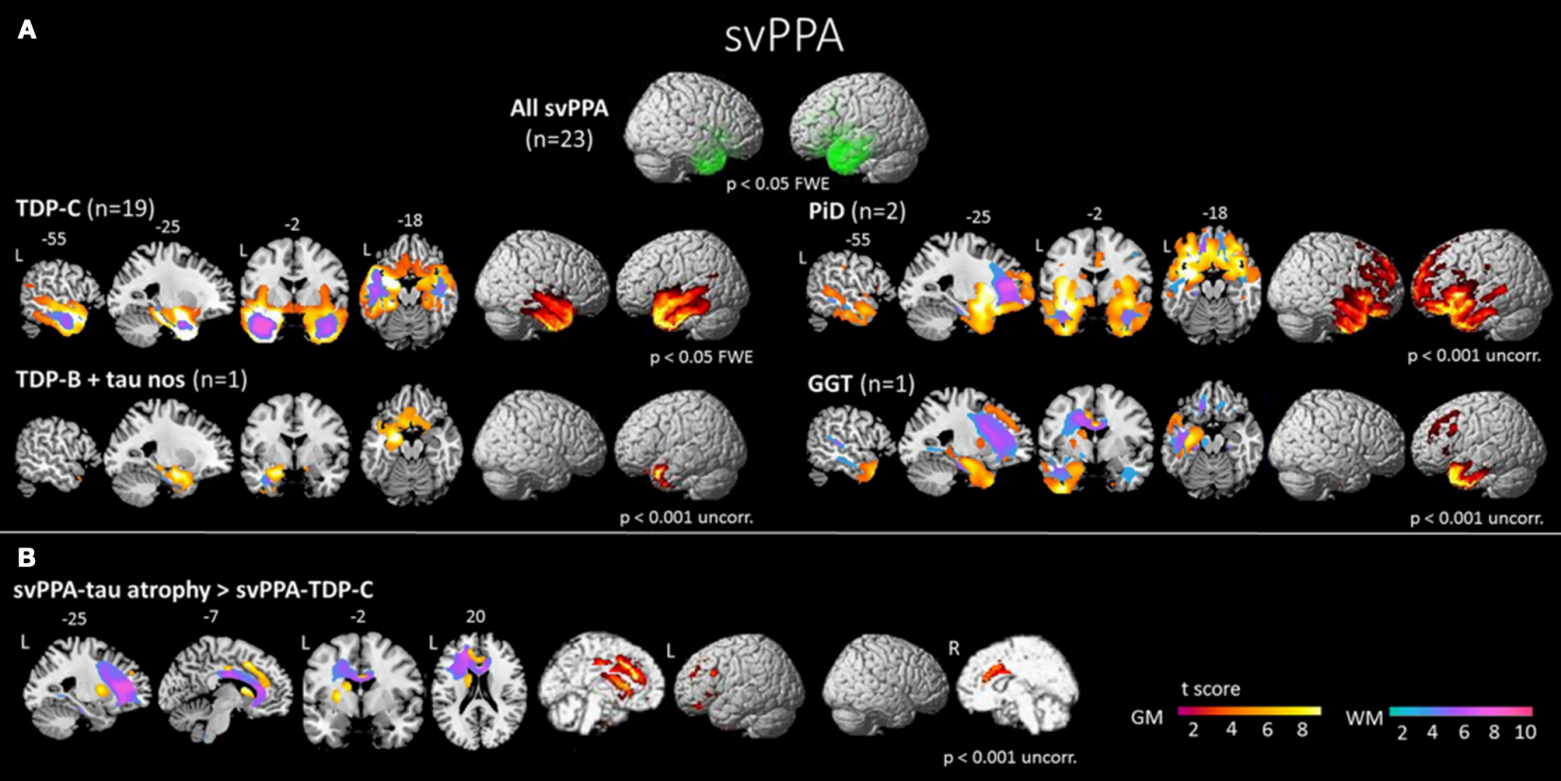
**(A)** Areas of significant atrophy in a group of patients with semantic variant of primary progressive aphasia (svPPA) and subdivided by pathological subgroups. The main areas of atrophy are in the bilateral anterior temporal lobes, more predominantly in the left hemisphere. **(B)** Areas of significant atrophy in svPPA patients with FTD tau pathology, in comparison to those with TDP-43 pathology. These patients present greater atrophy of frontotemporal cortex (medial anterior temporal lobe, orbitofrontal cortex, anterior cingulate cortex), basal ganglia and connecting white matter structures. FWE, family wise error; TDP-C, svPPA patients with FTD-TDP-43 depositions type C pathology; TDP-C+ tau nos, svPPA patients with FTD-TDP-43 depositions type C and tau pathology not otherwise specified; PiD, svPPA patients with Pick's disease pathology; GGT, svPPA patients with globular glial tauopathy pathology; L, left; R, right; GM, gray matter; WM, white matter [from (45), Permission to reproduce have been obtained from the copyright holders of this work].

The anterior temporal lobes show bilateral atrophy and hypoperfusion in svPPA and is considered as the syndrome-specific epicenter (45, 101–104) (Figure 4). This focal anatomical damage makes neuroimaging a complementary tool in the diagnostic process for this PPA variant (105). The damage is usually greater in the left hemisphere at first stages of the disease (30). Typical semantic impairment is associated with greater left-sided anterior temporal atrophy/hypometabolism (106, 107), naming difficulties are correlated with superior portions of the left temporal pole (108), and finally, loss of person knowledge and behavioral changes are associated with more extensive right temporal atrophy (109, 110). Atrophy of the hippocampus has been reported mainly involving the anterior portion, which is connected to the semantic memory system (111). On the other hand, the posterior portion, mainly connected to the episodic memory system, would be relatively spared (112). This pattern of hippocampal atrophy would explain the dissociation between semantic and episodic memory deficits in svPPA population.

Microstructural studies of WM integrity have shown damage in the ventral tracts that connect the temporal lobe to the occipital lobe and to the orbitofrontal cortex, with left side predominance (Figure 3) (51). The dorsal frontoparietal tracts that do not involve the temporal lobes are spared bilaterally, except for the temporal segment of the dorsal pathway (Figure 3) (51, 113, 114). This pattern is highly left lateralized compared with behavioral variant of fronto-temporal dementia which has a right predominance (115). During the progression of the disease, right-hemisphere WM bundles, in particular the uncinate, are preferentially damaged (116).

At the functional level, svPPA patients manifest extensive reduced anterior temporal lobe connectivity with primary and modality-selective cortices (117, 118). The longitudinal pattern of atrophy can be predicted by functional MRI connectivity between the temporal pole and the rest of the brain following connectional pathways within a large-scale network (119).

As the disease progresses, the atrophy involves the ventral and lateral temporal regions, as well as the contralateral temporal lobe and frontal regions (57, 120, 121). Beyond the specific metabolic signatures, additional dysfunctional patterns in the early stages can predict clinical progression: svPPA patients who present with extended bilateral patterns at baseline eventually develop behavioral disorders and a dysexecutive syndrome at follow-up (122).

#### Pathology

SvPPA is nearly always associated with underlying TDP-43-C pathological aggregates (75–100% in clinicopathological correlation series), and for the remainder of patients, most often with FTD tau (45, 60, 62, 66, 123–126). Rarely, AD pathology has also been reported in svPPA (60, 126), although a recent study has shown that most svPPA cases with AD pathology also present TDP-43-C pathology (68). In comparison to patients with TDP-43 pathology, those with FTD tau pathology present greater atrophy of frontotemporal cortex (medial anterior temporal lobe, orbitofrontal cortex, anterior cingulate cortex), basal ganglia and connecting WM bundles (Figure 4) (45). Interestingly, svPPA patients and PGRN mutation carriers are both characterized by underlying TDP-43 aggregation. In some cases, patients with PGRN mutations can develop aphasia with semantic deficits (127). However, familial forms of pure svPPA have not been reported. In both svPPA patients and PGRN mutation carriers, an increased prevalence of specific and related autoimmune diseases has been found, suggesting a unique pattern of systemic inflammation (128). Very rarely, mutations of the C9ORF72 gene have also been described in svPPA (129).

Recent studies have tried to identify FTD pathological subtypes with the help of cerebrospinal fluid (CSF) biomarkers. Increased neurofilament light chain (NfL) levels in the CSF, which are associated with neuronal and axonal degeneration, have been reported in patients with neurodegenerative diseases, and more specifically in patients with probable TDP-43 pathology such as svPPA patients (130).

### Logopenic variant (lvPPA)

#### Clinical manifestations

LvPPA has been more recently characterized (10, 131) as a distinct form of PPA, and little is known about its age at onset and disease survival. As with the other variants of PPA, the logopenic variant is considered an early onset form of dementia (132).

Patients with lvPPA typically present with word finding difficulty, along with sentence repetition deficits and, as the disease progresses, impaired sentence comprehension. Phonological impairments and, specifically, a phonological short-term memory deficit, have been suggested to be the core of the syndrome (131). In accordance with this interpretation, repetition and comprehension of single words remaining largely spared.

Prior to the current consensus criteria for diagnosis, lvPPA was often diagnosed as nfvPPA (60). Both variants can present with slow speech, frequent word-finding pauses and speech sound errors. However, patients with nfvPPA have slower speech and, conversely, those with lvPPA do not present with severe agrammatism and the distorted, effortful speech production of AOS. Speech sound errors are usually phonemic, but not phonetic (21, 23). Confrontation naming is often impaired, albeit at a lesser degree if compared to svPPA. Phonological paraphasias can occur in spontaneous speech and confrontation naming.

Usually later in the disease, episodic memory impairment (62) is often present, even though the lexical retrieval impairment observed in lvPPA patients contributes to verbal episodic memory performance (133, 134). Longitudinal studies have shown that cognitive decline is faster in lvPPA in comparison to other variants, and that this decline is not restricted to language functioning (135). In lvPPA patients, a more accelerated decline was also observed in visuospatial abilities (136), in memory and in attention (135). This finding has been associated with the underlying AD pathology (see section Pathology). Poor calculation abilities (137) and limb apraxia can also occur (10). Apathy, anxiety, irritability, and agitation are often reported (138).

Recent studies have revealed that in comparison to the general population, PPA patients report higher rates of learning disabilities (and more specifically developmental dyslexia) in their early phases of life (139), and that dyslexia susceptibility genes influence brain atrophy in PPA (140). Further reports suggested that the frequency was specifically higher in lvPPA patients in comparison to the other variants, and that in these patients, learning disability is associated with earlier onset of disease, more isolated language symptoms, and more focal pattern of left posterior temporoparietal atrophy (97). Developmental dyslexia, which is the most common developmental language learning disability, can manifest with phonological disturbances and posterior temporal involvement, similarly to lvPPA. In the framework of network vulnerability hypothesis, learning disability might confer susceptibility of language network to early-onset, focal AD pathology such as lvPPA (97). However, further research is needed to confirm the higher frequency of developmental dyslexia in lvPPA specifically, since another study provided conflicting results (141).

Criteria for lvPPA (11) require that both of the following core features must be present: impaired single-word retrieval in spontaneous speech and naming along with impaired repetition of sentences and phrases. At least 3 of the following other features must be present: (1) phonological errors in spontaneous speech and naming; (2) spared single-word comprehension and object knowledge; (3) spared motor speech; and (4) absence of frank agrammatism.

#### Linguistic/cognitive assessment

The evaluation of spontaneous speech is essential in order to appreciate lvPPA patients' anomia and can be done using description of a picture as previously described. In such tasks, anomia may manifest in phonological paraphasias, hesitations, and frequent pauses for word-finding. Language assessment of lvPPA also includes confrontation naming tasks such as the Boston Naming Test (98). Oral repetition of words, pseudowords, phrases, and sentences, are usually administered in order to show the dissociation between preserved single word repetition, in opposition to the greater impairment for sentences and phrases. Moreover, phonological errors are often appreciated (142). Tests for sentence comprehension consist of matching orally presented sentences to pictures. Patients with nfvPPA also fail on these types of tests because of the effect of grammatical complexity, whereas patients with lvPPA fail because of the effects of length and frequency.

Reading and spelling tests reveal phonological errors as well as difficulty with pseudowords, which rely upon phonological processing (91). LvPPA patients also show difficulties in verbal working memory tests, such as the digit span from the Weschler Adult Intelligence Scale (48, 143). Single-word comprehension, motor speech and agrammatism should be evaluated as exclusion criteria. Recent studies have suggested that non-linguistic features, namely visuospatial functioning (136), episodic memory and emotion processing (144), are also helpful in the differential diagnosis of lvPPA in comparison to the other variants (and specially with nfvPPA).

#### Neuroimaging findings

Anatomical damage in lvPPA is typically located in posterior superior temporal and middle temporal gyri as well as the inferior parietal lobule (10, 45). This pattern of atrophy is consistent with the classical anatomical model of the phonological loop (131). This neurodegenerative pattern is very similar to the one observed in early-onset AD (132). Naming difficulties are correlated with the left posterior temporal cortex (108). Recently, a model of the progression of atrophy in lvPPA has been suggested, showing that atrophy progresses from the disease epicenter (left posterior superior and middle temporal gyri) to ipsilateral parietal and frontal lobes and contralateral temporal lobe (145).

WM loss in association tracts in the left hemisphere has been detected (146), mainly in parietal fibers linking the parietal with frontal and posterior temporal regions (Figure 3) (51). A recent longitudinal study also suggested that early WM changes in lvPPA can be observed in the left posterior inferior longitudinal fasciculus, and that these changes become widespread over a year of progression of the disease, also affecting the anterior inferior longitudinal fasciculus, the uncinate fasciculus and the superior longitudinal fasciculus (116).

In terms of functional connectivity, the working memory network (frontal regions, inferior parietal lobule, superior, and middle temporal gyri) and language network (posterior superior temporal gyrus and inferior frontal lobe) have been shown to be altered in lvPPA patients in a resting-state fMRI study (147).

#### Pathology

LvPPA is most often caused by AD pathology (45, 60, 65), in as many as 95% of cases (68). It is considered one of the possible focal and early onset presentations of AD (62, 107, 132), even if other pathological profiles have been more rarely described, including Lewy body dementia (148), TDP-43, and tau (60).

**Figure 5:**
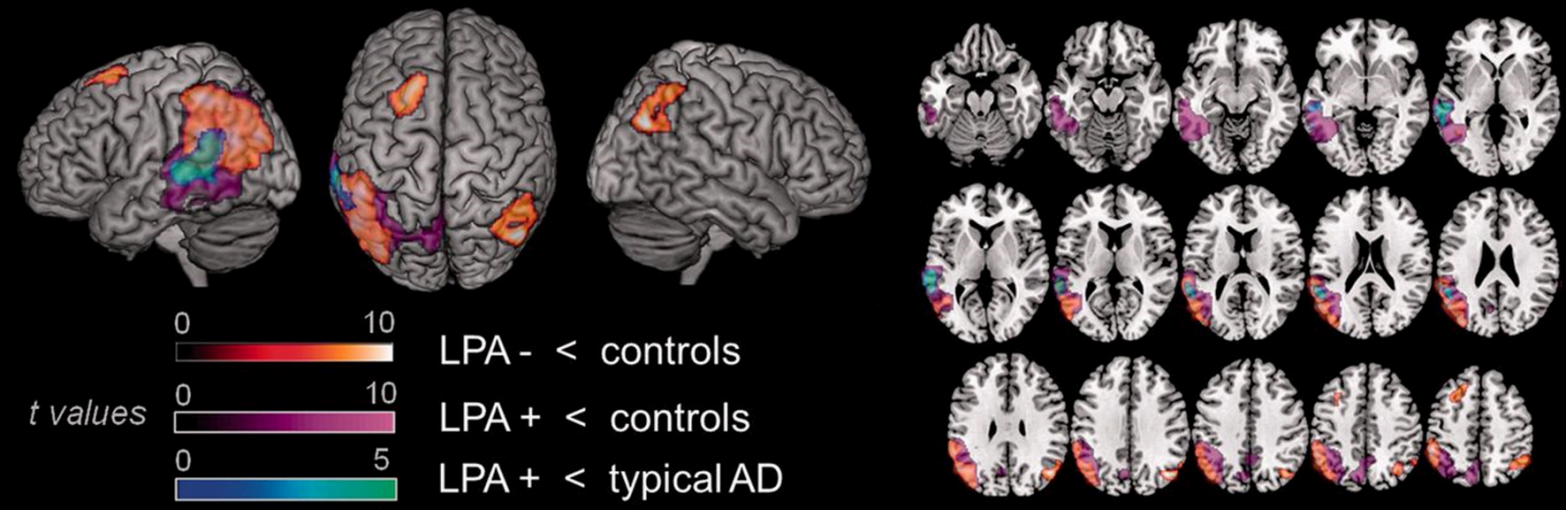
Comparison of areas of hypoperfusion between groups of lvPPA patients with AD pathology, lvPPA patients without AD pathology and typical AD patients. LvPPA patients present significant hypoperfusion in the left temporal-parietal junction, affecting larger portions of the temporal cortex in the lvPPA patients with AD pathology. In comparison to typical AD patients, lvPPA patients with AD pathology show more significant hypoperfusion in the left superior temporal gyrus. LPA, lvPPA; LPA-, lvPPA patients without AD pathology; LPA+, lvPPA patients with AD pathology; AD, Alzheimer's disease [from (149), Permission to reproduce have been obtained from the copyright holders of this work].

Cerebro-spinal fluid examination (149) and molecular imaging techniques such as PET with Pittsburgh Compound B (PIB) (107), a ligand for the amyloid, have shown the presence of amyloid in these individuals. However, in comparison to typical AD patients, lvPPA patients with AD pathology show more significant hypoperfusion in the left superior temporal gyrus (Figure 5) (149).

Some authors have demonstrated the coexistence of AD pathology and argyrophilic thorny astrocyte clusters (ATAC), intensely tau immunoreactive, in the fronto-temporoparietal cortex and subcortical regions (150). They suggest that they might represent a marker of a process responsible for the prominent focal clinical manifestations in lvPPA (150). Finally, a clinical syndrome with lvPPA features but also with more global features may be pre-dominantly linked to mutations in the GRN gene (151).

### PPA unclassifiable

In addition to the three most common variants, the 2011 consensus criteria also suggest that a minority of patients might be unclassifiable (11). It might be the case in patients who present for a long time with a single language symptom or in patients who present mixed features. It was further suggested that with the progression of the disease, these patients' profiles might become clearer.

A few studies have directly investigated the extent to which the 2011 consensus criteria cover the diversity of PPA cases. While some of them found that most patients could be classified within the three suggested variants (60, 61, 68, 152), others reported higher percentage (20–41%) of unclassifiable PPA patients (153–155). One of the main difficulty described by these last authors is related to patients who present with a single isolated language symptom, thus fulfilling the root criteria for PPA, but not the criteria for any of the variants. The most often reported cases are the ones who present with isolated anomia, without impaired repetition of single word, sentences and phrases (therefore only partially fulfilling criteria for lvPPA). Another main difficulty reported is related to patients who present a mixed profile, thus fulfilling the criteria for more than one of the variants. Patients presenting both sentence repetition impairments and agrammatism (therefore fulfilling criteria for both lvPPA and nfvPPA), as well as patients presenting both agrammatism and semantic impairments (fulfilling criteria for both nfvPPA and svPPA) have been observed.

Many factors could be associated with a varying proportion of unclassifiable PPA cases. First, the potential inclusion of genetic forms of PPA in studies might lead to higher number of mixed profiles. For example, it has been suggested that PGRN mutation carriers might present with a lvPPA and nfvPPA mixed profile (156). Furthermore, there is likely a high heterogeneity in tests and cutoffs used in the diagnosis of PPA across different clinical and research sites. There is a need for a use of sensitive and specific tests, and some authors have recently published assessment batteries specifically designed for the assessment of PPAs (157, 158). Most importantly, apparently similar symptoms might be due to distinct underlying causes between PPA variants, and this should be considered in tests selection and interpretation. For example, some studies have suggested that repetition deficits might be of different nature in lvPPA and nfvPPA. In lvPPA, they might be due to a disruption of the store component of the phonological loop, while in nfvPPA, impairments in speech motor planning might affect the subvocal rehearsal component of the phonological loop (159). This has also been investigated regarding the overlapping naming impairments in svPPA and lvPPA, which are thought to be respectively due to a semantic impairment vs. a lexical access deficit (160, 161). Finally, more prospective studies investigating the classification of PPA patients are needed, since retrospective studies are more likely to not have had adequate or complete test batteries to apply the current criteria (68). Nonetheless, even though significant progress was made in the recent years in the understanding of PPA, these studies illustrate that there are still controversies in the diagnosis of PPA and the above-mentioned issues will need to be clarified in the next years.

## Conclusion

In summary, each variant of PPA (nfv-, sv-, and lv-PPA) is characterized by a prototypic neurolinguistic/neuropsychological, neuroimaging and neuropathological profile. The effervescence of PPA as a research field in the recent years has allowed for key discoveries in each of these domains. In terms of clinical advancements, recent studies have allowed a better characterization and differentiation of PPA patients based on both their linguistic and non-linguistic profiles. In terms of neuroimaging, techniques such as diffusion imaging and resting-state fMRI, as well as multimodal studies, have allowed a deeper understanding of the impact of PPA on structural and functional connectivity alterations beyond the well-defined pattern of regional gray matter atrophy. Finally, in terms of pathology/genetics, despite significant advances, clinico-pathological correspondence is still far to be absolute. The improved characterization of PPA has the potential to have a positive impact on the management of individual patients. It can help to better direct patients toward appropriate therapeutic and behavioral intervention, as well as to provide adequate counseling of families and caregivers (162). Improved reliability of diagnoses and the development of reliable *in vivo* biomarkers for underlying neuropathology will be increasingly important as trials for etiology-specific treatments become available.

## Author contributions

MLG-T and RM contributed to the conception of the study. RM and MM wrote the first draft of the manuscript, but all authors wrote sections of the manuscript. All authors contributed to manuscript revision, read, and approved the submitted version.

### Conflict of interest statement

The authors declare that the research was conducted in the absence of any commercial or financial relationships that could be construed as a potential conflict of interest.

## Abbreviations

AD: Alzheimer's disease
AOS: apraxia of speech
ATAC: argyrophilic thorny astrocyte clusters
CBD: corticobasal degeneration
CBS: corticobasal syndrome
C9ORF72: chromosome 9 open reading frame 72
FTD: frontotemporal dementia
GM: gray matter
lvPPA: logopenic variant primary progressive aphasia
MAPT: microtubule-associated protein tau
MRI: magnetic resonance imaging
nfvPPA: non-fluent/agrammatic variant primary progressive aphasia
PET: positron emission tomography
PGRN: progranulin
PIB: Pittsburgh Compound B
PPA: primary progressive aphasia
PPAOS: progressive apraxia of speech
PSP: progressive supranuclear palsy
PSP-s: progressive supranuclear palsy syndrome
SMA: supplementary motor area
svPPA: semantic variant primary progressive aphasia
TDP-43: transactive response DNA-binding protein 43
WM: white matter.

## References

[1] Pick A Ueber die beziehungen der senilen hirnatrophie zur aphasie. Prager Med Wochenschrift (1892) 17:165–7.

[2] Sérieux P Déjèrine J Sur un cas de surdité verbale pure terminée par aphasie sensorielle, suivie d'autopsie. Comptes Rendues des Séances de la Soc Biol. (1897) 49:1074–7.

[3] Mesulam MM. Primary progressive aphasia: a 25-year retrospective. Alzheimer Dis Assoc Disord. (2007) 21:S8–11. 10.1097/WAD.0b013e31815bf7e1 18090430

[4] Mesulam MM. Slowly progressive aphasia without generalized dementia. Ann Neurol. (1982) 11:592–8. 10.1002/ana.410110607 7114808

[5] Warrington EK. The selective impairment of semantic memory. Q J Exp Psychol. (1975) 27:635–57. 10.1080/14640747508400525 1197619

[6] Snowden J Goulding PJ Neary D Semantic dementia: a form of circumscribed cerebral atrophy. Behav Neurol. (1989) 2:167–82.

[7] Hodges JR Patterson K Oxbury S Funnell E. Semantic dementia. Progressive fluent aphasia with temporal lobe atrophy. Brain (1992) 115(Pt 6):1783–806. 1486461 10.1093/brain/115.6.1783

[8] Grossman M Mickanin J Onishi K Progressive non-fluent aphasia: language, cognitive and PET measures contrasted with probable Alzheimer's disease. J Cogn Neurosci. (1996) 8:135–54. 23971420 10.1162/jocn.1996.8.2.135

[9] Neary D Snowden JS Gustafson L Passant U Stuss D Black S. Frontotemporal lobar degeneration: a consensus on clinical diagnostic criteria. Neurology (1998) 51:1546–54. 9855500 10.1212/wnl.51.6.1546

[10] Gorno-Tempini ML Dronkers NF Rankin KP Ogar JM Phengrasamy L Rosen HJ. Cognition and anatomy in three variants of primary progressive aphasia. Ann Neurol. (2004) 55:335–46. 10.1002/ana.10825 14991811 PMC2362399

[11] Gorno-Tempini ML Hillis AE Weintraub S Kertesz A Mendez M Cappa SF. Classification of primary progressive aphasia and its variants. Neurology (2011) 76:1006–14. 10.1212/WNL.0b013e31821103e6 21325651 PMC3059138

[12] Mesulam MM Primary progressive aphasia. Ann Neurol. (2001) 49:425–32. 10.1002/ana.91 11310619

[13] Seeley WW Crawford RK Zhou J Miller BL Greicius MD. Neurodegenerative diseases target large-scale human brain networks. Neuron (2009) 62:42–52. 10.1016/j.neuron.2009.03.024 19376066 PMC2691647

[14] Grundke-Iqbal I Iqbal K Quinlan M Tung YC Zaidi MS Wisniewski HM. Microtubule-associated protein tau. A component of Alzheimer paired helical filaments. J Biol Chem. (1986) 261:6084–9. 3084478

[15] Andreadis A Brown WM Kosik KS. Structure and novel exons of the human tau gene. Biochemistry (1992) 31:10626–33. 1420178 10.1021/bi00158a027

[16] Mackenzie IR Rademakers R Neumann M. TDP-43 and FUS in amyotrophic lateral sclerosis and frontotemporal dementia. Lancet Neurol. (2010) 9:995–1007. 10.1016/S1474-4422(10)70195-2 20864052

[17] Johnson JK Diehl J Mendez MF Neuhaus J Shapira JS Forman M. Frontotemporal lobar degeneration: demographic characteristics of 353 patients. Arch Neurol. (2005) 62:925–30. 10.1001/archneur.62.6.925 15956163

[18] Hodges JR Davies R Xuereb J Kril J Halliday G Survival in frontotemporal dementia. Neurology (2003) 61:349–54. 10.1212/01.WNL.0000078928.20107.52 12913196

[19] Josephs KA Knopman DS Whitwell JL Boeve BF Parisi JE Petersen RC. Survival in two variants of tau-negative frontotemporal lobar degeneration: FTLD-U vs FTLD-MND. Neurology (2005) 65:645–7. 10.1212/01.wnl.0000173178.67986.7f 16116138

[20] Ogar JM Dronkers NF Brambati SM Miller BL Gorno-Tempini ML. Progressive nonfluent aphasia and its characteristic motor speech deficits. Alzheimer Dis Assoc Disord. (2007) 21:S23–30. 10.1097/WAD.0b013e31815d19fe 18090419

[21] Ash S Evans E O'Shea J Powers J Boller A Weinberg D. Differentiating primary progressive aphasias in a brief sample of connected speech. Neurology (2013) 81:329–36. 10.1212/WNL.0b013e31829c5d0e 23794681 PMC3772830

[22] Ash S McMillan C Gunawardena D Avants B Morgan B Khan A. Speech errors in progressive non-fluent aphasia. Brain Lang. (2010) 113:13–20. 10.1016/j.bandl.2009.12.001 20074786 PMC2839014

[23] Wilson SM Henry ML Besbris M Ogar JM Dronkers NF Jarrold W. Connected speech production in three variants of primary progressive aphasia. Brain (2010) 133(Pt 7):2069–88. 10.1093/brain/awq129 20542982 PMC2892940

[24] Josephs KA Duffy JR Strand EA Whitwell JL Layton KF Parisi JE. Clinicopathological and imaging correlates of progressive aphasia and apraxia of speech. Brain (2006) 129(Pt 6):1385–98. 10.1093/brain/awl078 16613895 PMC2748312

[25] Thompson CK Mack JE. Grammatical Impairments in PPA. Aphasiology (2014) 28:1018–37. 10.1080/02687038.2014.912744 25642014 PMC4306464

[26] Hillis AE Oh S Ken L. Deterioration of naming nouns versus verbs in primary progressive aphasia. Ann Neurol. (2004) 55:268–75. 10.1002/ana.10812 14755731

[27] Rosen HJ Allison SC Ogar JM Amici S Rose K Dronkers N. Behavioral features in semantic dementia vs other forms of progressive aphasias. Neurology (2006) 67:1752–6. 10.1212/01.wnl.0000247630.29222.34 17130406

[28] Rohrer JD Rossor MN Warren JD. Syndromes of nonfluent primary progressive aphasia: a clinical and neurolinguistic analysis. Neurology (2010) 75:603–10. 10.1212/WNL.0b013e3181ed9c6b 20713949 PMC2931766

[29] Kertesz A Martinez-Lage P Davidson W Munoz DG. The corticobasal degeneration syndrome overlaps progressive aphasia and frontotemporal dementia. Neurology (2000) 55:1368–75. 10.1212/WNL.55.9.1368 11087783

[30] Gorno-Tempini ML Murray RC Rankin KP Weiner MW Miller BL. Clinical, cognitive and anatomical evolution from nonfluent progressive aphasia to corticobasal syndrome: a case report. Neurocase (2004) 10:426–36. 10.1080/13554790490894011 15788282 PMC2365737

[31] Murray R Neumann M Forman MS Farmer J Massimo L Rice A. Cognitive and motor assessment in autopsy-proven corticobasal degeneration. Neurology (2007) 68:1274–83. 10.1212/01.wnl.0000259519.78480.c3 17438218

[32] Deramecourt V Lebert F Debachy B Mackowiak-Cordoliani MA Bombois S Kerdraon O. Prediction of pathology in primary progressive language and speech disorders. Neurology (2010) 74:42–9. 10.1212/WNL.0b013e3181c7198e 19940270

[33] McMonagle P Blair M Kertesz A. Corticobasal degeneration and progressive aphasia. Neurology (2006) 67:1444–51. 10.1212/01.wnl.0000240215.43492.01 17060571

[34] Rohrer JD Paviour D Bronstein AM O'Sullivan SS Lees A Warren JD. Progressive supranuclear palsy syndrome presenting as progressive nonfluent aphasia: a neuropsychological and neuroimaging analysis. Mov Disord. (2010) 25:179–88. 10.1002/mds.22946 20077483 PMC4608044

[35] Armstrong MJ Litvan I Lang AE Bak TH Bhatia KP Borroni B. Criteria for the diagnosis of corticobasal degeneration. Neurology (2013) 80:496–503. 10.1212/WNL.0b013e31827f0fd1 23359374 PMC3590050

[36] Hoglinger GU Respondek G Stamelou M Kurz C Josephs KA Lang AE. Clinical diagnosis of progressive supranuclear palsy: The movement disorder society criteria. Mov Disord. (2017) 32:853–64. 10.1002/mds.26987 28467028 PMC5516529

[37] Goodglass H Kaplan E Boston Diagnostic Aphasia Examination (BDAE). Odessa, FL: Lea and Febiger Distributed by Psychological Assessment Resources (1983).

[38] Kertesz A Western Aphasia Battery. London, ON: University of Western Ontario Press (1980).

[39] Mayer M Frog, Where Are You?. New York, NY: Penguin Books (1969).

[40] Boschi V Catricalà E Consonni M Chesi C Moro A Cappa SF. Connected speech in neurodegenerative language disorders: a review. Front Psychol. (2017) 8:269. 10.3389/fpsyg.2017.00269 28321196 PMC5337522

[41] Wertz RT LaPointe LL Rosenbek JC Apraxia of Speech in Adults: The Disorder and its Management. Indiana: Singular (1984).

[42] Weintraub S Mesulam MM Wieneke C Rademaker A Rogalski EJ Thompson CK. The northwestern anagram test: measuring sentence production in primary progressive aphasia. Am J Alzheimers Dis Other Demen. (2009) 24:408–16. 10.1177/1533317509343104 19700669 PMC2836907

[43] Mandelli ML Vilaplana E Brown JA Hubbard HI Binney RJ Attygalle S. Healthy brain connectivity predicts atrophy progression in non-fluent variant of primary progressive aphasia. Brain (2016) 139(Pt 10):2778–91. 10.1093/brain/aww195 27497488 PMC5035819

[44] Mandelli ML Vitali P Santos M Henry M Gola K Rosenberg L. Two insular regions are differentially involved in behavioral variant FTD and nonfluent/agrammatic variant PPA. Cortex (2016) 74:149–57. 10.1016/j.cortex.2015.10.012 26673947 PMC4755480

[45] Spinelli EG Mandelli ML Miller ZA Santos-Santos MA Wilson SM Agosta F. Typical and atypical pathology in primary progressive aphasia variants. Ann Neurol. (2017) 81:430–43. 10.1002/ana.24885 28133816 PMC5421819

[46] Mandelli ML Caverzasi E Binney RJ Henry ML Lobach I Block N. Frontal white matter tracts sustaining speech production in primary progressive aphasia. J Neurosci. (2014) 34:9754–67. 10.1523/JNEUROSCI.3464-13.2014 25031413 PMC4099550

[47] Wilson SM Dronkers NF Ogar JM Jang J Growdon ME Agosta F. Neural correlates of syntactic processing in the nonfluent variant of primary progressive aphasia. J Neurosci. (2010) 30:16845–54. 10.1523/JNEUROSCI.2547-10.2010 21159955 PMC3024013

[48] Gorno-Tempini ML Ogar JM Brambati SM Wang P Jeong JH Rankin KP. Anatomical correlates of early mutism in progressive nonfluent aphasia. Neurology (2006) 67:1849–51. 10.1212/01.wnl.0000237038.55627.5b 16931509

[49] Josephs KA Duffy JR Strand EA Machulda MM Senjem ML Lowe VJ. Syndromes dominated by apraxia of speech show distinct characteristics from agrammatic PPA. Neurology (2013) 81:337–45. 10.1212/WNL.0b013e31829c5ed5 23803320 PMC3772832

[50] Wilson SM Galantucci S Tartaglia MC Rising K Patterson DK Henry ML. Syntactic processing depends on dorsal language tracts. Neuron (2011) 72:397–403. 10.1016/j.neuron.2011.09.014 22017996 PMC3201770

[51] Galantucci S Tartaglia MC Wilson SM Henry ML Filippi M Agosta F. White matter damage in primary progressive aphasias: a diffusion tensor tractography study. Brain (2011) 134(Pt 10):3011–29. 10.1093/brain/awr099 21666264 PMC3187537

[52] Grossman M Powers J Ash S McMillan C Burkholder L Irwin D. Disruption of large-scale neural networks in non-fluent/agrammatic variant primary progressive aphasia associated with frontotemporal degeneration pathology. Brain Lang. (2013) 127:106–20. 10.1016/j.bandl.2012.10.005 23218686 PMC3610841

[53] Grossman M. The non-fluent/agrammatic variant of primary progressive aphasia. Lancet Neurol. (2012) 11:545–55. 10.1016/S1474-4422(12)70099-6 22608668 PMC3361730

[54] Bonakdarpour B Rogalski EJ Wang A Sridhar J Mesulam MM Hurley RS Functional Connectivity is Reduced in Early-stage Primary Progressive Aphasia When Atrophy is not Prominent. Alzheimer Dis Assoc Disord. (2017) 31:101–6. 10.1097/wad.0000000000000193 28288010 PMC5443692

[55] Rogalski E Cobia D Harrison TM Wieneke C Weintraub S Mesulam MM. Progression of language decline and cortical atrophy in subtypes of primary progressive aphasia. Neurology (2011) 76:1804–10. 10.1212/WNL.0b013e31821ccd3c 21606451 PMC3100122

[56] Santos-Santos MA Mandelli ML Binney RJ Ogar J Wilson SM Henry ML Features of patients with nonfluent/agrammatic primary progressive aphasia with underlying progressive supranuclear palsy pathology or corticobasal degeneration. JAMA Neurol. (2016) 73:733–42. 10.1001/jamaneurol.2016.0412 27111692 PMC4924620

[57] Brambati SM Amici S Racine CA Neuhaus J Miller Z Ogar J. Longitudinal gray matter contraction in three variants of primary progressive aphasia: a tenser-based morphometry study. Neuroimage Clin. (2015) 8:345–55. 10.1016/j.nicl.2015.01.011 26106560 PMC4473099

[58] Catani M Mesulam MM Jakobsen E Malik F Martersteck A Wieneke C. A novel frontal pathway underlies verbal fluency in primary progressive aphasia. Brain (2013) 136(Pt 8):2619–28. 10.1093/brain/awt163 23820597 PMC3722349

[59] Josephs KA Hodges JR Snowden JS Mackenzie IR Neumann M Mann DM. Neuropathological background of phenotypical variability in frontotemporal dementia. Acta Neuropathol. (2011) 122:137–53. 10.1007/s00401-011-0839-6 21614463 PMC3232515

[60] Chare L Hodges JR Leyton CE McGinley C Tan RH Kril JJ. New criteria for frontotemporal dementia syndromes: clinical and pathological diagnostic implications. J Neurol Neurosurg Psychiatry (2014) 85:865–70. 10.1136/jnnp-2013-306948 24421286

[61] Harris JM Gall C Thompson JC Richardson AM Neary D du Plessis D. Classification and pathology of primary progressive aphasia. Neurology (2013) 81:1832–9. 10.1212/01.wnl.0000436070.28137.7b 24142474

[62] Mesulam M Wicklund A Johnson N Rogalski E Leger GC Rademaker A. Alzheimer and frontotemporal pathology in subsets of primary progressive aphasia. Ann Neurol. (2008) 63:709–19. 10.1002/ana.21388 18412267 PMC2858311

[63] Mesulam MM Weintraub S Rogalski EJ Wieneke C Geula C Bigio EH. Asymmetry and heterogeneity of Alzheimer's and frontotemporal pathology in primary progressive aphasia. Brain (2014) 137(Pt 4):1176–92. 10.1093/brain/awu024 24574501 PMC3959558

[64] Rohrer JD Lashley T Schott JM Warren JE Mead S Isaacs AM. Clinical and neuroanatomical signatures of tissue pathology in frontotemporal lobar degeneration. Brain (2011) 134(Pt 9):2565–81. 10.1093/brain/awr198 21908872 PMC3170537

[65] Rohrer JD Rossor MN Warren JD. Alzheimer's pathology in primary progressive aphasia. Neurobiol Aging (2012) 33:744–52. 10.1016/j.neurobiolaging.2010.05.020 20580129 PMC3314936

[66] Snowden J Neary D Mann D. Frontotemporal lobar degeneration: clinical and pathological relationships. Acta Neuropathol. (2007) 114:31–8. 10.1007/s00401-007-0236-3 17569065

[67] Cioffi SM Galimberti D Barocco F Spallazzi M Fenoglio C Serpente M. Non fluent variant of primary progressive aphasia due to the novel GRN g.9543delA(IVS3-2delA) mutation. J Alzheimers Dis. (2016) 54:717–21. 10.3233/jad-160185 27567822

[68] Santos-Santos MA Rabinovici GD Iaccarino L. Rates of amyloid imaging positivity in patients with primary progressive aphasia. JAMA Neurol. (2018) 75:342–52. 10.1001/jamaneurol.2017.4309 29309493 PMC5885868

[69] Caso F Mandelli ML Henry M Gesierich B Bettcher BM Ogar J. *In vivo* signatures of nonfluent/agrammatic primary progressive aphasia caused by FTLD pathology. Neurology (2014) 82:239–47. 10.1212/WNL.0000000000000031 24353332 PMC3902758

[70] Snowden J Goulding PJ Neary D Semantic dementia: a form of circumscribed cerebral atrophy. Behav Neurol. (1989) 2:167–82

[71] Hodges JR Patterson K. Semantic dementia: a unique clinicopathological syndrome. Lancet Neurol. (2007) 6:1004–14. 10.1016/S1474-4422(07)70266-1 17945154

[72] Kertesz A Blair M McMonagle P Munoz DG. The diagnosis and course of frontotemporal dementia. Alzheimer Dis Assoc Disord. (2007) 21:155–63. 10.1097/WAD.0b013e31806547eb 17545742

[73] Kertesz A Jesso S Harciarek M Blair M McMonagle P. What is semantic dementia?: a cohort study of diagnostic features and clinical boundaries. Arch Neurol. (2010) 67:483–9. 10.1001/archneurol.2010.55 20385916

[74] Coyle-Gilchrist IT Dick KM Patterson K Vazquez Rodriquez P Wehmann E Wilcox A. Prevalence, characteristics, and survival of frontotemporal lobar degeneration syndromes. Neurology (2016) 86:1736–43. 10.1212/wnl.0000000000002638 27037234 PMC4854589

[75] Caine D Breen N Patterson K. Emergence and progression of ‘non-semantic' deficits in semantic dementia. Cortex (2009) 45:483–94. 10.1016/j.cortex.2007.07.005 19231477

[76] Garrard P Rentoumi V Gesierich B Miller B Gorno-Tempini ML. Machine learning approaches to diagnosis and laterality effects in semantic dementia discourse. Cortex (2014) 55:122–9. 10.1016/j.cortex.2013.05.008 23876449 PMC4072460

[77] Mayberry EJ Sage K Ralph MA At the edge of semantic space: the breakdown of coherent concepts in semantic dementia is constrained by typicality and severity but not modality. J Cogn Neurosci. (2011) 23:2240–51. 10.1162/jocn.2010.21582 21126159

[78] Hoffman P Lambon Ralph MA Reverse concreteness effects are not a typical feature of semantic dementia: evidence for the hub-and-spoke model of conceptual representation. Cereb Cortex (2011) 21:2103–12. 10.1093/cercor/bhq288. 21285258

[79] Yi HA Moore P Grossman M. Reversal of the concreteness effect for verbs in patients with semantic dementia. Neuropsychology (2007) 21:9–19. 10.1037/0894-4105.21.1.9 17201526

[80] Joubert S Vallet GT Montembeault M Boukadi M Wilson MA Laforce RJ. Comprehension of concrete and abstract words in semantic variant primary progressive aphasia and Alzheimer's disease: a behavioral and neuroimaging study. Brain Lang. (2017) 170:93–102. 10.1016/j.bandl.2017.04.004 28432988

[81] Gainotti G. Different patterns of famous people recognition disorders in patients with right and left anterior temporal lesions: a systematic review. Neuropsychologia (2007) 45:1591–607. 10.1016/j.neuropsychologia.2006.12.013 17275042

[82] Mendez MF Kremen SA Tsai PH Shapira JS. Interhemispheric differences in knowledge of animals among patients with semantic dementia. Cogn Behav Neurol. (2010) 23:240–6. 10.1097/WNN.0b013e3181f22448 21042206 PMC3143503

[83] Josephs KA Whitwell JL Vemuri P Senjem ML Boeve BF Knopman DS. The anatomic correlate of prosopagnosia in semantic dementia. Neurology (2008) 71:1628–33. 10.1212/01.wnl.0000334756.18558.92 19001253 PMC2676968

[84] Mondini S Semenza C. How Berlusconi keeps his face: a neuropsychological study in a case of semantic dementia. Cortex (2006) 42:332–5. 10.1016/S0010-9452(08)70359-9 16771038

[85] Irish M Bunk S Tu S Kamminga J Hodges JR Hornberger M. Preservation of episodic memory in semantic dementia: the importance of regions beyond the medial temporal lobes. Neuropsychologia (2016) 81:50–60. 10.1016/j.neuropsychologia.2015.12.005 26683384

[86] Adlam AL Patterson K Hodges JR “I remember it as if it were yesterday”: memory for recent events in patients with semantic dementia. Neuropsychologia (2009) 47:1344–51. 10.1016/j.neuropsychologia.2009.01.029 19428398

[87] Irish M Hornberger M Lah S Miller L Pengas G Nestor PJ. Profiles of recent autobiographical memory retrieval in semantic dementia, behavioural-variant frontotemporal dementia, and Alzheimer's disease. Neuropsychologia (2011) 49:2694–702. 10.1016/j.neuropsychologia.2011.05.017 21658396

[88] Viard A Desgranges B Matuszewski V Lebreton K Belliard S de La Sayette V. Autobiographical memory in semantic dementia: new insights from two patients using fMRI. Neuropsychologia (2013) 51:2620–32. 10.1016/j.neuropsychologia.2013.08.007 23954715

[89] Irish M Addis DR Hodges JR Piguet O. Considering the role of semantic memory in episodic future thinking: evidence from semantic dementia. Brain (2012) 135(Pt 7):2178–91. 10.1093/brain/aws119 22614246

[90] La Corte V Piolino P On the Role of Personal semantic memory and temporal distance in episodic future thinking: the TEDIFT model. Front Hum Neurosci. (2016) 10:385 10.3389/fnhum.2016.00385 27524964 PMC4965476

[91] Brambati SM Ogar J Neuhaus J Miller BL Gorno-Tempini ML. Reading disorders in primary progressive aphasia: a behavioral and neuroimaging study. Neuropsychologia (2009) 47:1893–900. 10.1016/j.neuropsychologia.2009.02.033 19428421 PMC2734967

[92] Wilson SM Brambati SM Henry RG Handwerker DA Agosta F Miller BL. The neural basis of surface dyslexia in semantic dementia. Brain (2009) 132(Pt 1):71–86. 10.1093/brain/awn300 19022856 PMC2638692

[93] Irish M Hodges JR Piguet O. Right anterior temporal lobe dysfunction underlies theory of mind impairments in semantic dementia. Brain (2014) 137(Pt 4):1241–53. 10.1093/brain/awu003 24523434

[94] Seeley WW Bauer AM Miller BL Gorno-Tempini ML Kramer JH Weiner M. The natural history of temporal variant frontotemporal dementia. Neurology (2005) 64:1384–90. 10.1212/01.WNL.0000158425.46019.5C 15851728 PMC2376750

[95] Kumfor F Miller L Lah S Hsieh S Savage S Hodges JR. Are you really angry? The effect of intensity on facial emotion recognition in frontotemporal dementia. Soc Neurosci. (2011) 6:502–14. 10.1080/17470919.2011.620779 21957889

[96] Gan JJ Lin A Samimi MS Mendez MF. Somatic symptom disorder in semantic dementia: the role of alexisomia. Psychosomatics (2016) 57:598–604. 10.1016/j.psym.2016.08.002 27647568

[97] Miller ZA Mandelli ML Rankin KP Henry ML Babiak MC Frazier DT. Handedness and language learning disability differentially distribute in progressive aphasia variants. Brain (2013) 136(Pt 11):3461–73. 10.1093/brain/awt242 24056533 PMC3808687

[98] Kaplan E Goodglass H Weintraub S Boston Naming Test: Philadelphia, PA: Lea & Febiger (1983).

[99] Howard D Patterson K The Pyramids and Palm Trees Test. A Test of Semantic Access From Words and Pictures. Bury St. Edmunds: Thames Valley Company (1992).

[100] Montembeault M Brambati SM Joubert S Boukadi M Chapleau M Laforce RJ. Naming unique entities in the semantic variant of primary progressive aphasia and Alzheimer's disease: Towards a better understanding of the semantic impairment. Neuropsychologia (2017) 95:11–20. 10.1016/j.neuropsychologia.2016.12.009 27939367

[101] Mummery CJ Patterson K Price CJ Ashburner J Frackowiak RS Hodges JR. A voxel-based morphometry study of semantic dementia: relationship between temporal lobe atrophy and semantic memory. Ann Neurol. (2000) 47:36–45. 10.1002/1531-8249(200001)47:1<36::AID-ANA8>3.0.CO;2-L 10632099

[102] Galton CJ Patterson K Graham K Lambon-Ralph MA Williams G Antoun N. Differing patterns of temporal atrophy in Alzheimer's disease and semantic dementia. Neurology (2001) 57:216–25. 10.1212/WNL.57.2.216 11468305

[103] Mesulam M Wieneke C Rogalski E Cobia D Thompson C Weintraub S. Quantitative template for subtyping primary progressive aphasia. Arch Neurol. (2009) 66:1545–51. 10.1001/archneurol.2009.288 20008661 PMC2796598

[104] Rosen HJ Kramer JH Gorno-Tempini ML Schuff N Weiner M Miller BL. Patterns of cerebral atrophy in primary progressive aphasia. Am J Geriatr Psychiatry (2002) 10:89–97 11790639

[105] Yang J Pan P Song W Shang HF. Quantitative meta-analysis of gray matter abnormalities in semantic dementia. J Alzheimers Dis. (2012) 31:827–33. 10.3233/jad-2012-120736 22699847

[106] Kato T Inui Y Nakamura A Ito K. Brain fluorodeoxyglucose (FDG) PET in dementia. Ageing Res Rev. (2016) 30:73–84. 10.1016/j.arr.2016.02.003 26876244

[107] Rabinovici GD Jagust WJ Furst AJ Ogar JM Racine CA Mormino EC. Abeta amyloid and glucose metabolism in three variants of primary progressive aphasia. Ann Neurol. (2008) 64:388–401. 10.1002/ana.21451 18991338 PMC2648510

[108] Migliaccio R Boutet C Valabregue R Ferrieux S Nogues M Lehéricy S. The brain network of naming: a lesson from primary progressive aphasia. PLoS ONE (2016) 11:e0148707. 10.1371/journal.pone.0148707 26901052 PMC4764674

[109] Thompson SA Patterson K Hodges JR. Left/right asymmetry of atrophy in semantic dementia: behavioral-cognitive implications. Neurology (2003) 61:1196–203. 10.1212/01.WNL.0000091868.28557.B8 14610120

[110] Henry ML Wilson SM Ogar JM Sidhu MS Rankin KP Cattaruzza T. Neuropsychological, behavioral, and anatomical evolution in right temporal variant frontotemporal dementia: a longitudinal and post-mortem single case analysis. Neurocase (2012) 20:100–9. 10.1080/13554794.2012.732089 23171151 PMC3775867

[111] La Joie R Landeau B Perrotin A Bejanin A Egret S Pelerin A Intrinsic connectivity identifies the hippocampus as a main crossroad between Alzheimer's and semantic dementia-targeted networks. Neuron (2014) 81:1417–28. 10.1016/j.neuron.2014.01.026 24656258

[112] Chapleau M Aldebert J Montembeault M Brambati SM. Atrophy in Alzheimer's disease and semantic dementia: an ALE meta-analysis of voxel-based morphometry studies. J Alzheimers Dis. (2016) 54:941–55. 10.3233/jad-160382 27567843

[113] Agosta F Henry RG Migliaccio R Neuhaus J Miller BL Dronkers NF. Language networks in semantic dementia. Brain (2010) 133(Pt 1):286–99. 10.1093/brain/awp233 19759202 PMC2801321

[114] Acosta-Cabronero J Patterson K Fryer TD Hodges JR Pengas G Williams GB. Atrophy, hypometabolism and white matter abnormalities in semantic dementia tell a coherent story. Brain (2011) 134(Pt 7):2025–35. 10.1093/brain/awr119 21646331

[115] Meijboom R Steketee RM Ham LS van der Lugt A van Swieten JC Smits M. Differential hemispheric predilection of microstructural white matter and functional connectivity abnormalities between respectively semantic and behavioral variant frontotemporal dementia. J Alzheimers Dis. (2017) 56:789–804. 10.3233/jad-160564 28059782

[116] Tu S Leyton CE Hodges JR Piguet O Hornberger M. Divergent longitudinal propagation of white matter degradation in logopenic and semantic variants of primary progressive aphasia. J Alzheimers Dis. (2016) 49:853–61. 10.3233/jad-150626 26484929

[117] Guo CC Gorno-Tempini ML Gesierich B Henry M Trujillo A Shany-Ur T. Anterior temporal lobe degeneration produces widespread network-driven dysfunction. Brain (2013) 136(Pt 10):2979–91. 10.1093/brain/awt222 24072486 PMC3857932

[118] Agosta F Galantucci S Valsasina P Canu E Meani A Marcone A. Disrupted brain connectome in semantic variant of primary progressive aphasia. Neurobiol Aging (2014) 35:2646–55. 10.1016/j.neurobiolaging.2014.05.017 24970567

[119] Collins JA Montal V Hochberg D Quimby M Mandelli ML Makris N. Focal temporal pole atrophy and network degeneration in semantic variant primary progressive aphasia. Brain (2017) 140(Pt 2):457–71. 10.1093/brain/aww313 28040670 PMC5278308

[120] Brambati SM Rankin KP Narvid J Seeley WW Dean D Rosen HJ. Atrophy progression in semantic dementia with asymmetric temporal involvement: a tensor-based morphometry study. Neurobiol Aging (2009) 30:103–11. 10.1016/j.neurobiolaging.2007.05.014 17604879 PMC2643844

[121] Kumfor F Landin-Romero R Devenney E Hutchings R Grasso R Hodges JR. On the right side? A longitudinal study of left- versus right-lateralized semantic dementia. Brain (2016) 139(Pt 3):986–98. 10.1093/brain/awv387 26811253

[122] Cerami C Dodich A Greco L Iannaccone S Magnani G Marcone A. The role of single-subject brain metabolic patterns in the early differential diagnosis of primary progressive aphasias and in prediction of progression to dementia. J Alzheimers Dis. (2016) 55:183–97. 10.3233/JAD-160682 27662315 PMC5115609

[123] Hodges JR Davies RR Xuereb JH Casey B Broe M Bak TH. Clinicopathological correlates in frontotemporal dementia. Ann Neurol. (2004) 56:399–406. 10.1002/ana.20203 15349867

[124] Davies RR Hodges JR Kril JJ Patterson K Halliday GM Xuereb JH. The pathological basis of semantic dementia. Brain (2005) 128(Pt 9):1984–95. 10.1093/brain/awh582 16000337

[125] Grossman M Wood EM Moore P Neumann M Kwong L Forman MS. TDP-43 pathologic lesions and clinical phenotype in frontotemporal lobar degeneration with ubiquitin-positive inclusions. Arch Neurol. (2007) 64:1449–54. 10.1001/archneur.64.10.1449 17923628

[126] Hodges JR Mitchell J Dawson K Spillantini MG Xuereb JH McMonagle P. Semantic dementia: demography, familial factors and survival in a consecutive series of 100 cases. Brain (2010) 133(Pt 1):300–6. 10.1093/brain/awp248 19805492

[127] Mesulam M Johnson N Krefft TA Gass JM Cannon AD Adamson JL. Progranulin mutations in primary progressive aphasia: the PPA1 and PPA3 families. Arch Neurol. (2007) 64:43–7. 10.1001/archneur.64.1.43 17210807

[128] Miller ZA Rankin KP Graff-Radford NR Takada LT Sturm VE Cleveland CM. TDP-43 frontotemporal lobar degeneration and autoimmune disease. J Neurol Neurosurg Psychiatry (2013) 84:956–62. 10.1136/jnnp-2012-304644 23543794 PMC3840954

[129] Le Ber I Camuzat A Guillot-Noel L Hannequin D Lacomblez L Golfier V C9ORF72 repeat expansions in the frontotemporal dementias spectrum of diseases: a flow-chart for genetic testing. J Alzheimers Dis. (2013) 34:485–99. 10.3233/jad-121456 23254636

[130] Scherling CS Hall T Berisha F Klepac K Karydas A Coppola G. Cerebrospinal fluid neurofilament concentration reflects disease severity in frontotemporal degeneration. Ann Neurol. (2014) 75:116–26. 10.1002/ana.24052 24242746 PMC4020786

[131] Gorno-Tempini ML Brambati SM Ginex V Ogar J Dronkers NF Marcone A. The logopenic/phonological variant of primary progressive aphasia. Neurology (2008) 71:1227–34. 10.1212/01.wnl.0000320506.79811.da 18633132 PMC2676989

[132] Migliaccio R Agosta F Rascovsky K Karydas A Bonasera S Rabinovici GD. Clinical syndromes associated with posterior atrophy: early age at onset AD spectrum. Neurology (2009) 73:1571–8. 10.1212/WNL.0b013e3181c0d427 19901249 PMC2777069

[133] Win KT Pluta J Yushkevich P Irwin DJ McMillan CT Rascovsky K. Neural correlates of verbal episodic memory and lexical retrieval in logopenic variant primary progressive aphasia. Front Neurosci. (2017) 11:330. 10.3389/fnins.2017.00330 28659753 PMC5469881

[134] Win KT Pluta J Yushkevich P Irwin DJ McMillan CT Rascovsky K. Neural correlates of verbal episodic memory and lexical retrieval in logopenic variant primary progressive aphasia. Front Neurosci. (2017) 11:330. 10.3389/fnins.2017.00330. 28659753 PMC5469881

[135] Leyton CE Hsieh S Mioshi E Hodges JR. Cognitive decline in logopenic aphasia: more than losing words. Neurology (2013) 80:897–903. 10.1212/WNL.0b013e318285c15b 23390170

[136] Watson CL Possin K Allen IE Hubbard HI Meyer M Welch AE. Visuospatial functioning in the primary progressive aphasias. J Int Neuropsychol Soc. (2017):24:259–68. 10.1017/S1355617717000984. 29039275 PMC5820206

[137] Rohrer JD Ridgway GR Crutch SJ Hailstone J Goll JC Clarkson MJ. Progressive logopenic/phonological aphasia: erosion of the language network. Neuroimage (2010) 49:984–93. 10.1016/j.neuroimage.2009.08.002 19679189 PMC2943046

[138] Rohrer JD Warren JD. Phenomenology and anatomy of abnormal behaviours in primary progressive aphasia. J Neurol Sci. (2010) 293:35–8. 10.1016/j.jns.2010.03.012 20400120 PMC2896484

[139] Rogalski E Johnson N Weintraub S Mesulam M. Increased frequency of learning disability in patients with primary progressive aphasia and their first-degree relatives. Arch Neurol. (2008) 65:244–8. 10.1001/archneurol.2007.34 18268195 PMC2892116

[140] Paternicó D Premi E Alberici A Archetti S Bonomi E Gualeni V. Dyslexia susceptibility genes influence brain atrophy in frontotemporal dementia. Neurol Genet (2015) 1:e24. 10.1212/NXG.0000000000000024 27066561 PMC4809460

[141] Rogalski E Rademaker A Wieneke C Bigio E Weintraub S Mesulam MM The prevalence of learning disabilities in primary progressive aphasia is not segregated by pathology or subtype. JAMA Neurol. (2014) 71:1576–7. 10.1001/jamaneurol.2014.2805 25486208 PMC4283581

[142] Leyton CE Ballard KJ Piguet O Hodges JR. Phonologic errors as a clinical marker of the logopenic variant of PPA. Neurology (2014) 82:1620–7. 10.1212/wnl.0000000000000387 24706011

[143] Wechsler D The Wechsler Adult Intelligence Scale–Revised. San Antonio, TX: Psychological Corporation (1981).

[144] Piguet O Leyton CE Gleeson LD Hoon C Hodges JR. Memory and emotion processing performance contributes to the diagnosis of non-semantic primary progressive aphasia syndromes. J Alzheimers Dis. (2015) 44:541–7. 10.3233/jad-141854 25298199

[145] Phillips JS Da Re F Dratch L Xie SX Irwin DJ McMillan CT. Neocortical origin and progression of gray matter atrophy in nonamnestic Alzheimer's disease. Neurobiol Aging (2018) 63:75–87. 10.1016/j.neurobiolaging.2017.11.008 29223682 PMC5801003

[146] Migliaccio R Agosta F Possin KL Rabinovici GD Miller BL Gorno-Tempini ML. White matter atrophy in Alzheimer's disease variants. Alzheimer's Dement. (2012) 8(Suppl. 5):S78–87e1-2. 10.1016/j.jalz.2012.04.010 23021625 PMC3717610

[147] Whitwell JL Jones DT Duffy JR Strand EA Machulda MM Przybelski SA. Working memory and language network dysfunctions in logopenic aphasia: a task-free fMRI comparison with Alzheimer's dementia. Neurobiol Aging (2015) 36:1245–52. 10.1016/j.neurobiolaging.2014.12.013 25592958 PMC4346438

[148] Teichmann M Migliaccio R Kas A Dubois B. Logopenic progressive aphasia beyond Alzheimer's–an evolution towards dementia with Lewy bodies. J Neurol Neurosurg Psychiatry (2013) 84:113–4. 10.1136/jnnp-2012-302638 22967721

[149] Teichmann M Kas A Boutet C Ferrieux S Nogues M Samri D. Deciphering logopenic primary progressive aphasia: a clinical, imaging and biomarker investigation. Brain (2013) 136(Pt 11):3474–88. 10.1093/brain/awt266 24108322

[150] Munoz DG Woulfe J Kertesz A. Argyrophilic thorny astrocyte clusters in association with Alzheimer's disease pathology in possible primary progressive aphasia. Acta Neuropathol. (2007) 114:347–57. 10.1007/s00401-007-0266-x 17637999

[151] Rohrer JD Warren JD Omar R Mead S Beck J Revesz T. Pariet al lobe deficits in frontotemporal lobar degeneration caused by a mutation in the progranulin gene. Arch Neurol. (2008) 65:506–13. 10.1001/archneur.65.4.506 18413474 PMC2578869

[152] Leyton CE Villemagne VL Savage S Pike KE Ballard KJ Piguet O. Subtypes of progressive aphasia: application of the International Consensus Criteria and validation using beta-amyloid imaging. Brain (2011) 134(Pt 10):3030–43. 10.1093/brain/awr216 21908392

[153] Mesulam MM Wieneke C Thompson C Rogalski E Weintraub S. Quantitative classification of primary progressive aphasia at early and mild impairment stages. Brain (2012) 135(Pt 5):1537–53. 10.1093/brain/aws080 22525158 PMC3577099

[154] Sajjadi SA Patterson K Arnold RJ Watson PC Nestor PJ. Primary progressive aphasia: a tale of two syndromes and the rest. Neurology (2012) 78:1670–7. 10.1212/WNL.0b013e3182574f79 22573633 PMC3359509

[155] Wicklund MR Duffy JR Strand EA Machulda MM Whitwell JL Josephs KA. Quantitative application of the primary progressive aphasia consensus criteria. Neurology (2014) 82:1119–26. 10.1212/wnl.0000000000000261 24598709 PMC3966800

[156] Rohrer JD Crutch SJ Warrington EK Warren JD. Progranulin-associated primary progressive aphasia: a distinct phenotype? Neuropsychologia (2010) 48:288–97. 10.1016/j.neuropsychologia.2009.09.017 19766663 PMC2808475

[157] Catricalà E Gobbi E Battista P Miozzo A Polito C Boschi V. SAND: a Screening for aphasia in neurodegeneration. development and normative data. Neurol Sci. (2017) 38:1469–83. 10.1007/s10072-017-3001-y 28578483

[158] Savage S Hsieh S Leslie F Foxe D Piguet O Hodges JR. Distinguishing subtypes in primary progressive aphasia: application of the Sydney language battery. Dement Geriatr Cogn Disord. (2013) 35:208–18. 10.1159/000346389 23467307

[159] Leyton CE Savage S Irish M Schubert S Piguet O Ballard KJ. Verbal repetition in primary progressive aphasia and Alzheimer's disease. J Alzheimers Dis. (2014) 41:575–85. 10.3233/jad-132468 24662100

[160] Leyton CE Hodges JR Piguet O Ballard KJ. Common and divergent neural correlates of anomia in amnestic and logopenic presentations of Alzheimer's disease. Cortex (2017) 86:45–54. 10.1016/j.cortex.2016.10.019 27875715

[161] Reilly J Peelle JE Antonucci SM Grossman M. Anomia as a marker of distinct semantic memory impairments in Alzheimer's disease and semantic dementia. Neuropsychology (2011) 25:413–26. 10.1037/a0022738 21443339 PMC3125450

[162] Galimberti D Cioffi SM Fenoglio C Serpente M Oblak AL Rodriguez-Porcel F. Rapidly progressive primary progressive aphasia and parkinsonism with novel GRN mutation. Mov Disord. (2017) 32:476–8. 10.1002/mds.26872 27859661

